# Leaf Litter Mixtures Alter Microbial Community Development: Mechanisms for Non-Additive Effects in Litter Decomposition

**DOI:** 10.1371/journal.pone.0062671

**Published:** 2013-04-29

**Authors:** Samantha K. Chapman, Gregory S. Newman, Stephen C. Hart, Jennifer A. Schweitzer, George W. Koch

**Affiliations:** 1 Department of Biology, Villanova University, Villanova, Pennsylvania, United States of America; 2 Department of Ecology and Evolutionary Biology, University of Tennessee, Knoxville, Tennessee, United States of America; 3 School of Natural Sciences, University of California Merced, Merced, California, United States of America; 4 Department of Biological Sciences, Northern Arizona University, Flagstaff, Arizona, United States of America; Wageningen University, Netherlands

## Abstract

To what extent microbial community composition can explain variability in ecosystem processes remains an open question in ecology. Microbial decomposer communities can change during litter decomposition due to biotic interactions and shifting substrate availability. Though relative abundance of decomposers may change due to mixing leaf litter, linking these shifts to the non-additive patterns often recorded in mixed species litter decomposition rates has been elusive, and links community composition to ecosystem function. We extracted phospholipid fatty acids (PLFAs) from single species and mixed species leaf litterbags after 10 and 27 months of decomposition in a mixed conifer forest. Total PLFA concentrations were 70% higher on litter mixtures than single litter types after 10 months, but were only 20% higher after 27 months. Similarly, fungal-to-bacterial ratios differed between mixed and single litter types after 10 months of decomposition, but equalized over time. Microbial community composition, as indicated by principal components analyses, differed due to both litter mixing and stage of litter decomposition. PLFA biomarkers *a*15∶0 and *cy*17∶0, which indicate gram-positive and gram-negative bacteria respectively, in particular drove these shifts. Total PLFA correlated significantly with single litter mass loss early in decomposition but not at later stages. We conclude that litter mixing alters microbial community development, which can contribute to synergisms in litter decomposition. These findings advance our understanding of how changing forest biodiversity can alter microbial communities and the ecosystem processes they mediate.

## Introduction

Changes in plant community structure, either through species loss or gain, can alter ecosystem processes such as litter decomposition through mechanisms that are poorly understood [1], [2]. Heterotrophic microbial decomposer communities closely track plant substrate availability and respond to changes in plant identity [3]–[5]. Microbial succession, defined here as a change in the abundance of certain functional decomposer groups on a single substrate over time, has been documented repeatedly on single plant litter types during decomposition [6]–[11]. Studies have shown that relative abundances of (1) bacteria vs. fungi (indicated by fungi-to-bacteria ratios; [12]–[14]); and (2) functional groups of decomposers can shift during decomposition of single litter types [13], [15]–[18]. However, plant litter types are almost always mixed in natural ecosystems, and little is known about how microbial communities change when realistic mixtures of leaf litter are decomposed *in situ* (but see [2], [3]). Mixing litter may cause shifts in microbial communities that result in differential decomposition dynamics, such as litter decay or mineral nitrogen (N) immobilization or release. Understanding microbial control of mixed litter decomposition may allow us to better predict carbon and nitrogen cycling as plant community structure changes.

Mixing of leaf litters often causes non-additive decomposition rates, in which the litter mixture decomposes at a rate not predictable by the decomposition rates of component litter types [19]–[24]. Hypotheses proposed to explain these synergistic (e.g., enhanced rates of decay) or antagonistic (e.g., slowed rates of decay) effects include litter environment and morphology [25], litter quality and nutrient transfer, [23], [26]–[30], microbial community and functional changes [3], [4], [19], [31], [32] and macrofaunal shifts [33]–[36]. Whole microbial community dynamics during mixed litter decomposition are important to explore in this context and have not been adequately assessed, but are indicative of the role microbial community composition may play in driving ecosystem functioning [8], [13].

Recent empirical studies have found that changing microbial community composition can have important impacts on ecosystem processes such as litter decomposition [37], [38]. Most studies on microbial community development during leaf litter decomposition have focused on bacterial and fungal decomposer abundance and identity [9]–[10], [15], [17], [39]. Shifts from bacterial-dominated to fungal-dominated decomposition have been observed [40], especially over short (days to a few months) time periods [10], [14]. These bacterial to fungal shifts, indicated by an increasing fungal-to-bacterial ratio, may be driven by gradually declining soluble carbon (C) compound availability, necessitating production of fungal enzymes that break down more complex C compounds [41]. Measurements of the relative abundances of decomposers over time scales relevant to terrestrial ecosystem carbon and nutrient cycling, which are usually longer than a few weeks, are rare [2], [16], [42], [43]. In a four-month study Wilkinson [13] found that bacterial biomass was initially higher than fungal biomass on spruce litter in Germany and both types of decomposers increased through time. In a one-year study Torres et al. [16] found that populations of ammonifying bacteria and sugar fungi (Zygomycetes) were stable throughout the study period, likely due to consistent N and soluble C availability from both litter decomposition and microbial turnover. Availability of soluble and recalcitrant compounds to decomposers likely differs due to litter mixing, thereby potentially altering bacterial and fungal abundances and litter decomposition rates [23].

We examined microbial community development on single and mixed species leaf litters in a long-term field study. We extracted phospholipid fatty acids (PLFAs) from single and mixed litterbags after 10 and 27 months of field decomposition in a high elevation mixed conifer forest. Previously in this system, we found that mixing leaf litter caused synergisms (or positive, non-additive effects) in decomposition rates (up to 50% increases; [22]). We also found that mixing similar litters (conifers) led to synergisms in litter decomposition and mixing litters of disparate chemical quality (conifers and aspen) did not [22]. After 10 months of litter decomposition, microbial diversity increased with increasing plant litter diversity [4]. Here, we build on this previous work to examine the links between microbial community development (over two years) and litter decomposition rate. The litter in this mixed conifer forest is fairly recalcitrant and has low nutrient concentrations, thus mixing litter may provide decomposers with different resources and facilitate a faster progression from decomposition of soluble compounds to predominantly recalcitrant compounds. We hypothesized that: 1) leaf litter mixing would increase abundance of fungal and bacterial decomposers as compared to single litters, 2) decomposer communities on both single and mixed litter would shift from bacterial-dominated decomposition (at 10 months) to fungal-dominated decomposition (at 27 months) as indicated by increasing fungal:bacterial (F:B) biomass ratios, 3) litter mixing and stage of decomposition would both alter microbial community structure, as indicated by principle components analyses (PCA) and 4) total PLFA and fungal-to-bacterial biomass ratios would correlate positively with increasing mass loss of both single and mixed species litter, indicating increasing decomposition with more microbial biomass and a shift towards fungal decomposers.

## Methods

### Experimental Design

We examined the impacts of mixing leaf litter on microbial dynamics in a mixed conifer forest on the San Francisco Peaks, 30 km north of Flagstaff, AZ (35.19N, −111.66W). All necessary permits were obtained from the Coconino National Forest for the described field studies. Details of the study site and litter decomposition studies are described in detail in Chapman and Koch [22]. Briefly, we decomposed leaf litter in single-tree species (monoculture) and mixed-tree species litterbags at a single site in six 25×25 m mixed-species blocks that were randomly located within a 0.5 km^2^ area with the same elevation (3050 m) and slope. Dominant tree species included *Populus tremuloides* Michx (quaking aspen; abbreviated as A), *Pseudotsuga menziesii* Mirbel Franco (Douglas-fir; D), *Pinus flexilis* James (limber pine; L), and *Pinus ponderosa* P. and C. Lawson (ponderosa pine; P). These four species range widely in litter C:N, lignin:N and leaf morphology, therefore providing diverse substrates and habitat for microbial communities [22]. Carbon to N ratios and lignin:N ratios were 70.8 and 11.5, respectively, in aspen litter, 45.4 and 13.5 in Douglas-fir, 46.7 and 17.8 in limber pine, and 71.3 and 25.9 in ponderosa pine (Table 1); [4].

**Table 1:** Litter chemical quality.

	Average carbon:nitrogen in litter	Average lignin:nitrogen in litter
**Aspen**	70.8	11.5
**Douglas Fir**	45.4	13.5
**Limber Pine**	46.7	17.8
**Ponderosa Pine**	71.3	25.9

Average carbon to nitrogen and lignin to nitrogen ratios of the four plant species litter types used in this experiment.

Leaf litter from the forest canopy for all four species was collected from buckets randomly placed in all six plots. Litter was sorted and bulked by species. A total of 11 litterbag types including each individual species (A, D, L, P), each pair-wise combination (A+D, A+L, A+P, D+L, D+P, L+P) and all four species (A+D+L+P) were made by weighing two grams of air-dried litter into 10×10 cm mesh bags. The mesh on the upper side of the bag was 0.8 mm polyester (Nylon Net, Memphis, TN) and allows access to soil organisms and the bottom mesh was 0.2 mm polypropylene mesh (Synthetic Industries, Atlanta, GA) to prevent loss. Though mesh bags can alter field litter decomposition rates [44], we are most interested in relative differences between the litter treatments and thus used this common technique. The initial mass of individual species’ litter placed in mixed species litterbags was equal to 2 g (air-dried) divided by the number of species (i.e., there was an equal total mass of litter in each bag). Litter decomposition bags were placed on the soil surface at a randomly chosen common location within each of the six larger 25×25 m plots. We replicated each ‘common litterbag garden’ three times within a plot for three successive removal periods: after 3, 10, and 27 months in the field. We did not measure litter PLFA at the outset of the experiment. PLFA analyses were only conducted on the 10- and 27-month litterbag removals.

At each collection date, the contents of the litterbags were carefully removed and dried at 70°C before weighing to assess mass loss. For the purposes of this study, mass loss was calculated as the average mass lost from the total contents (all component litter types). See [4], [22] for additional details.

### PLFA Extraction from Litter

Individual litter components were sorted by species according to morphological differences from all mixture litterbags. In order to obtain enough litter for extraction of each litter type (1 g fresh weight), we systematically bulked individual species litter from two litterbags from two different plots of the same mixture (for example we added aspen litter from one plot’s litter bag to aspen litter from another plot’s litter bag). We consistently bulked litter from the same plots over the two time intervals. This generated three replicate samples for each litter mixture from the six total plots. Litter was ground to a fine powder using a ball mill grinder (Model 2601, Cianflone Scientific Instruments, Pittsburgh, PA, USA). Phospholipid fatty acids were extracted from the freeze-dried litter with a phosphate-buffered chloroform-methanol solvent (1 g litter: 6 mL buffer plus 15 mL methanol and 7.5 mL chloroform [45], [46]). After methylation of the polar lipids, signature fatty acids were separated and analyzed by gas chromatography and mass spectrometry ([47]; Agilent Technologies GC-Mass Spectrometer [6890N GC/5973N MSD] Palo Alto, CA, USA). Different PLFAs are unique to different taxonomic groups (e.g., gram positive and gram negative bacteria, and fungi; [47]–[49]). While PLFAs provide a coarse measure of microbial community composition and abundance data need to be interpreted with caution [50], [51], evidence suggests PLFA analysis is often as effective in detecting treatment differences than both functional analyses and molecular techniques [52]. Though a total of 43 compounds were identified from the extractions, we limited our analyses to the 23 compounds between carbon chain lengths C14 and C19 to conservatively constrain our analyses to those known with relative certainty to be microbial [13], [42], [48], [50], [53]. Total PLFA concentration was calculated as the sum of all 23 compounds and can be used as an index of total microbial biomass [13], [40]. Fungal PLFA concentration was calculated as the sum of C18∶2n6t and C18∶2n6c [50]. The concentration of bacterial PLFA was calculated with the sum of the following compounds: *i*15∶0, *a*15∶0, C15∶0, *i*16∶0, C16∶1ω9, C16∶1, *cy*17∶0, C17∶0, and *cy*19∶0 [13], [42], [48], [50], [53], [54]. We used all 23 compounds in our PCA community analyses at the 10-month and 27-month harvest dates.

In order to obtain a value of PLFA biomass for mixed litterbags, we averaged the PLFA concentrations of the individual species litter components (which were extracted separately) from each bag. For example, PLFA concentration for the aspen-limber pine litter bag were obtained by averaging the aspen PLFA concentration and limber pine PLFA concentration obtained from that bag. This approach may not perfectly represent the actual PLFA concentration of a given mixture because litter decomposition may proceed faster or slower for each species, thereby creating a situation where each litter may not be half of the total mass. However, we could not obtain accurate masses for each species by the end of the experiment because much of the litter was significantly decomposed rendering it unidentifiable. Therefore, we could not perform a weighed average of PLFA concentrations. Though weighted averages would be ideal for understanding PLFA concentrations on mixed litter bags, obtaining this information was logistically impossible. However, in some cases, the sampling method we used may actually provide a conservative assessment of PLFA concentrations since conifer litter, which decomposes more slowly than aspen litter, tends to show larger increases of total PLFA from single to mixed litters.

### Data Analysis

**Figure 1:**
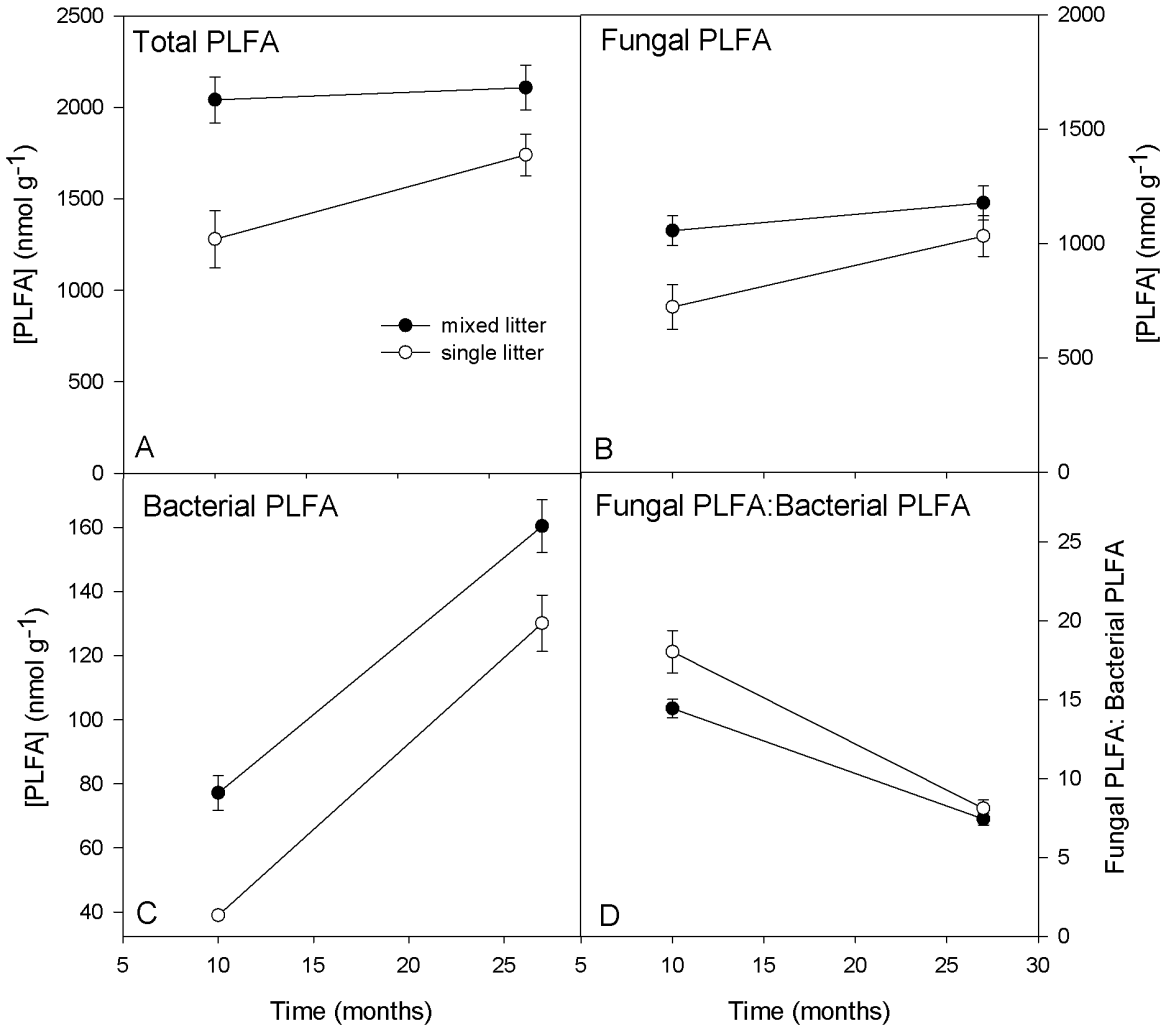
Microbial decomposer biomass on single and mixed leaf litter. The development of microbial communities on single and mixed litter types during leaf litter decomposition. In panels A and B, mixed litterbags had significantly higher total and fungal PLFA concentrations than single litterbags over the two litterbag harvest dates (p<0.01 in both cases). In panel C, bacterial PLFA changed significantly through time on single vs. mixed species litterbags time (p = 0.05). In panel D, there was a significant interaction between mixing effect and time for fungal:bacterial ratios (p = 0.01). Standard errors are indicated by bars on each point.

We examined how litter mixing impacted total, fungal, and bacterial PLFA biomass and fungal PLFA to bacterial PLFA ratio using repeated measures MANOVA with replicate, mixed vs. single litter treatments, and time as factors (Fig. 1). Though we analyzed all mixtures (2- and 4-species together for most analyses), we did examine whether these two mixture types differed from each other using one-way ANOVA at each time point. Microbial community structure was characterized by performing principle components analysis (PCA) on log_10_-transformed mole percentages of the 23 PLFA biomarkers. We subsequently analyzed the influence of litter mixing (single or mixed litters) and stage of decomposition (10 months and 27 months) on the first two principle component scores (compiled from all 23 biomarkers) using two-way ANOVA. We examined how litter mixing and stage of decomposition affected individual PLFA biomarkers (mole %) using two-way ANOVA with mixed vs. single litter treatments and harvest as factors. We used standard linear regressions to investigate correlations between different fungal and bacterial PLFA concentrations, fungal: bacterial ratio and litter decomposition. We calculated percent synergism of litter decomposition by subtracting the mean expected mass loss of a leaf litter mixture type from the mean observed mass loss of that leaf litter mixture type and dividing that difference by the mean expected mass loss of that litter mixture. We calculated the percent stimulation of PLFA biomass concentrations by performing the same calculations on PLFA observed and expected biomass of mixed litterbags. All statistical analyses were run using JMP 9 (SAS Institute, Cary, NC) and we set α = 0.05 for all analyses.

## Results

### Total, Fungal and Bacterial Biomass on Litter Mixtures vs. Single litters

Litter in mixed litterbags had significantly higher total PLFA concentrations than single species litterbags over the course of the experiment (repeated measures model p = 0.002, mixing effect p = 0.008; Figure 1A). There was no significant interaction between time and mixing effect; p = 0.15). Fungal PLFA was also higher on mixed litter than single species litterbags (repeated measures model p = 0.004, mixing effect p = 0.04, interaction p = 0.24; Figure 1B). Bacterial PLFA changed significantly through time on single vs. mixed species litterbags (model p = 0.048, time p<0.0001; Figure 1C). There was a significant interaction between time and mixing effect for F:B ratios in that as time progressed these ratios converged for mixed and single litter (interaction p = 0.01); Figure 1D). After ten months, two-species mixtures had lower total, fungal, and bacterial PLFA (p<0.01 for all three parameters), and lower F:B ratios, than four-species mixtures. After 27 months of decomposition, only bacterial PLFA differed between two-species mixtures and four-species mixtures (higher on 4-species mixtures; p = 0.01).

### Treatment Effects on Microbial Community Composition and Specific PLFAs

**Figure 2:**
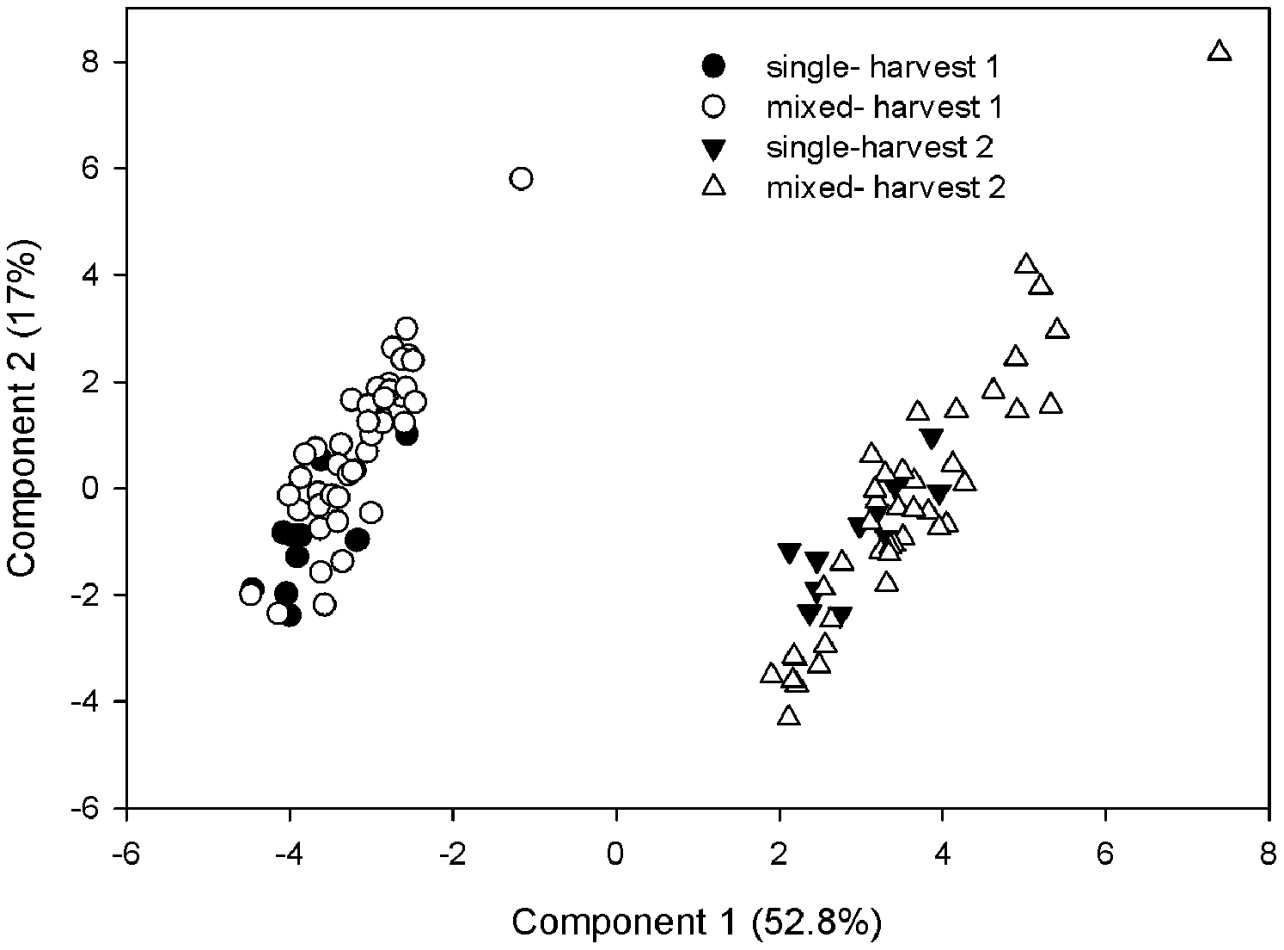
Litter microbial community composition changes due to mixing litter and stage of decomposition. Principle components analyses of PLFA profiles (log_10_ transformed mol%) on litter at two stages of decomposition (after 10 and 27 months in the field). Open symbols indicate single litterbags and solid symbols indicate mixed litterbags. Circles indicate the litterbags removed after 10 months and triangles indicate the litterbags removed after 27 months. Principle component (PC1) score was different between the two decomposition harvests (p<0.001) and between mixed and single litter (p<0.01). PC2 was significantly different for single litter vs. mixed litter (p = 0.01) but not between 10 and 27 months of decomposition.

All 23 PLFA biomarkers from each stage of decomposition (or harvest) for mixed and single litter types were analyzed using PCA. The resulting PC1 and PC2 had eigenvectors of 12.14 and 3.91 and explained 53% and 17% of total variation, respectively. PC scores separated out these data by harvest (see Fig. 2), but also by litter treatment (mixed or single treatments). The two-way ANOVA on PC1 scores showed significant treatment effects of both harvest (p<0.0001) and litter treatment (p = 0.01) however there was no significant interaction between the two factors. The two-way ANOVA on PC2 scores showed a significant effect of litter mixing (p = 0.01) but not of harvest or an interaction between litter treatment and harvest.

Specific PLFA biomarkers also changed due to treatment and through time. The biomarker *a*15∶0, which is often attributed to gram-positive bacteria, was higher on litter mixtures than litter monocultures and was lower on litter after 27 months than litter at 10 months (model p = 0.0003, mixture effect p = 0.001, harvest p = 0.001). A two-way ANOVA on *Cy*17∶0, which often indicates gram-negative bacteria, yielded a significant interaction between mixture effect and harvest (model p<0.0001, interaction p = 0.05). Fungal marker 18∶2n6t was higher on litter at 27 months than at 10 months (model p<0.0001, harvest p<0.0001) but the fungal marker 18∶2n6c did not change due to stage of decomposition.

### PLFA Biomass and Litter Decomposition

**Figure 3:**
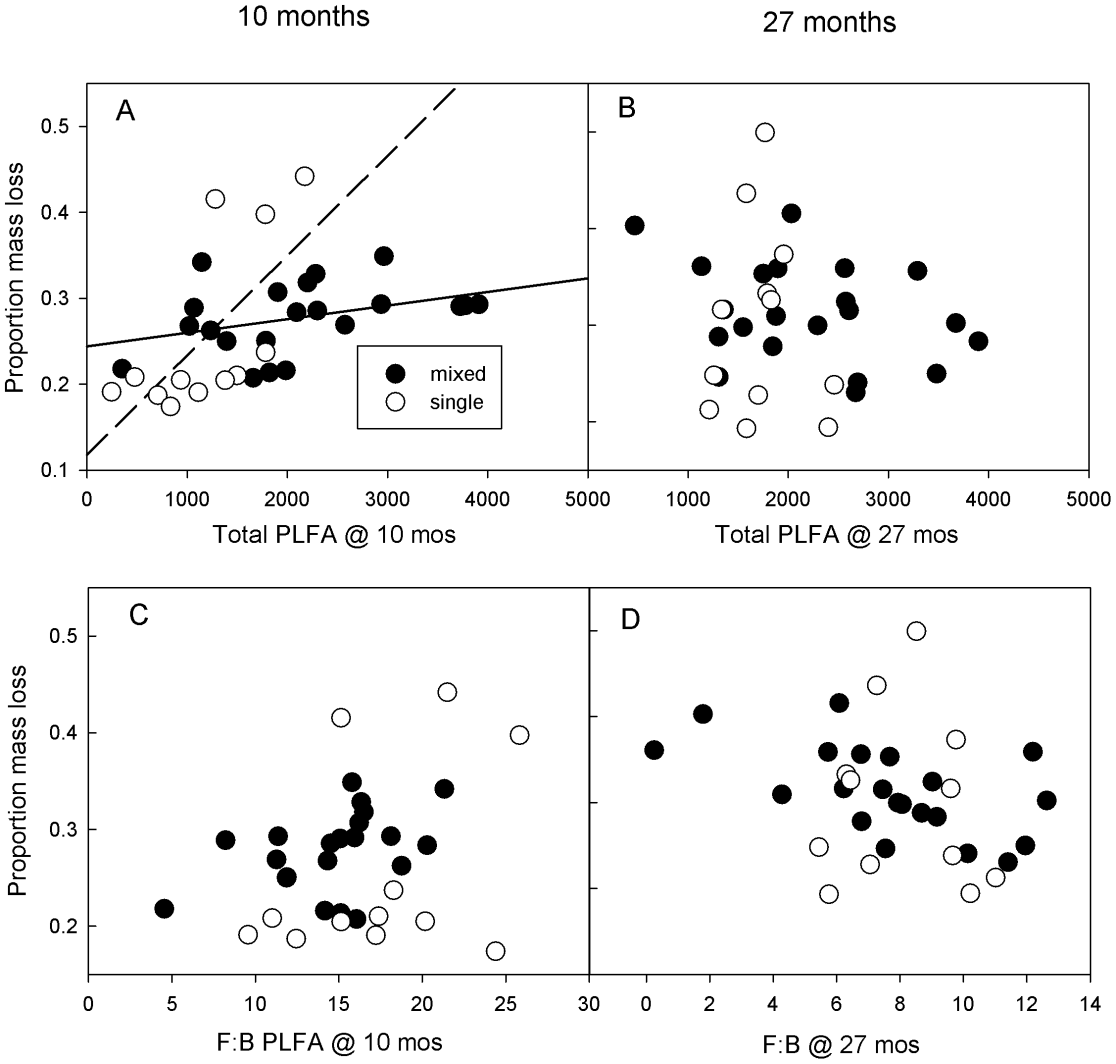
Microbial decomposer biomass and litter decomposition. Correlations between PLFA and litter decomposition for mixed (solid symbols, solid lines) and single litter types (open symbols, dashed lines). After 10 months of decomposition, total PLFA concentration significantly correlated with single litter decomposition (Pearsons coefficient (PC) = 0.67, p = 0.02) and tended to correlate with mixed litter decomposition though this correlation was not significant (PC = 0.33, p = 0.15; Panel A). There were no significant correlations between total PLFA concentration and litter decomposition at 27 months (Panel B). Fungal: bacterial ratios of PLFA showed a trend towards correlating with mixed litter decomposition at 10 months (PC = 0.36, p = 0.10) and 27 months (PC = 0.33, p = 0.14; Panel D) but not single litter decomposition at either time point.

Total PLFA (i.e. microbial biomass) correlates with higher mass loss in single litter treatments at 10 months (Pearsons correlation coefficient (PC) = 0.67, p = 0.02) but not after 27 months (Figure 3). Total PLFA did not correlate with mass loss for mixed litter treatments at 10 months (mix- PC = 0.33, p = 0.15) or after 27 months (Figure 3). Fungal to bacterial ratio of PLFA on mixed litter treatments did not correlate significantly with litter mass loss at 10 months (PC = 0.36, p = 0.10) or 27 months (PC = 0.33 p = 0.14) and did not correlate with mass loss of litter monocultures at either time point (Figure 3).

### Timing of Stimulations in Biomass and Litter Decomposition

Table 2 shows that percent stimulation of litter mass loss (above expected values) due to mixing is highest on average after 3 months, followed by the 10 month decomposition bag harvest date, while percent stimulation of PLFA is highest after 10 months in the field; PLFA were not extracted after 3 month harvest date. Regressions between percent stimulations and time couldn’t be performed because we only had two points of PLFA extraction (after 10 and 27 months). However, this table qualitatively shows that stimulations of both decomposition and PLFA concentrations due to litter mixing were highest during the early stages of litter decomposition.

**Table 2:** Observed and expected litter total PLFA, mass loss proportion and percent synergisms.

	Littertype	3 months			10 months					27 months		
		Obs.	Exp.	% syn.	Obs.		Exp.		% syn.	Obs.	Exp.	% syn.
**Mass loss proportion**	**AD**	0.24	0.24	2.3	0.32	0.31		4.0		0.36	0.35	1.7
	**AP**	0.21	0.23	−7.4	0.29	0.30		−4.8		0.32	0.32	−0.3
	**LA** **DL** **PD** **PL** **ALL**	0.25	0.22	11.6	0.33	0.32		2.5		0.36	0.37	−2.4
		0.17	0.12	48.8	0.25	0.21		22.3		0.31	0.28	10.7
		0.15	0.12	21.5	0.21	0.19		10.2		0.26	0.23	14.0
		0.16	0.11	47.2	0.26	0.20		27.8		0.29	0.25	15.4
		0.19	0.17	7.8	0.28	0.26		10.7		0.30	0.30	0.0
**Total PLFA (nmol g^−1^)**	**AD**				2281.9	1109.8		105.6		1778.1	1601.8	11.7
	**AP**				2250.2	1425.7		57.8		2167.0	1751.7	23.7
	**LA** **DL** **PD** **PL** **ALL**	Not measured			1821.8	1574.5		15.7		2884.7	1898.5	52.0
					1682.4	940.3		78.9		1972.3	1730.1	14.0
					1891.6	791.5		138.9		1564.6	1583.4	−1.2
					1266.1	1256.2		0.8		1615.3	1880.0	−14.1
					1922.4	1183.0		91.3		2437.5	1740.9	40.1

Mixing leaf litter often stimulated litter PLFA concentrations and mass loss above expected values, resulting in synergisms in many cases. Percent synergisms (% syn.) were determined by subtracting observed (shown as “Obs.) values from expected (shown as “Exp.”) values, dividing by expected values, and multiplying by 100. Litter types are indicated by the letters of the component species (A = aspen, D = Douglas fir, L = limber pine, P = Ponderosa pine, ALL- includes all four species).

## Discussion

We analyzed how microbial community changes on single and mixed species leaf litters during decomposition using a field experiment followed by PLFA analyses. In support of our first hypothesis, we found that total microbial biomass (as indicated by total, fungal and bacterial PLFA concentrations) was 70% higher on litter mixtures than single litter types after about 1 year but was only 20% higher after two years (Fig. 1). In a previous study, we suggested that microbial communities colonized mixed litter more rapidly due to the increased diversity of niches and substrates, as evidenced by higher fungal and bacterial colonization of mixed litter at 10 months ([4]; Fig. 1). This more rapid development may be indicated by a lower fungal-to-bacterial ratio, which is perhaps characteristic of a later stage of decomposition in this ecosystem since fungal-to-bacterial ratios progress to lower values (Fig. 1). Various researchers found that increasing litter chemical diversity correlated with increased soil respiration rates, perhaps due to complementarity effects [23], [32]. Similarly, the chemical diversity present in litter mixtures (Table 1) may allow more functionally even (bacteria and fungi) microbial communities to exist on mixed litter. This idea may be further supported by our finding that 4-species mixtures had higher total PLFA and lower F:B ratios on average than 2-species mixtures. It is also important to note that the variation in total PLFA concentrations is larger for mixtures than for any of the litter monocultures (Fig. 3). Litter mixtures containing high-quality aspen litter supported higher amounts of PLFA biomass, likely contributing to this variation (Table 2). Larger amounts of bacterial biomass were supported by mixed litter after twenty-seven months of litter decomposition. Kominoski et al. [3] found that mixing litter stimulated litter bacterial biomass above the expected amounts early in decomposition but not at later stages. Our findings also suggest that microbial biomass are equalized at later stages of decomposition, as indicated by the equivalent total and fungal PLFA concentrations on mixed and single litters after 27 months. If single litter microbial communities “catch up” to mixed litter communities, and differences in microbial communities cause synergisms in litter decomposition, this could help explain why we did not see pronounced litter synergisms at later stages of decomposition (Table 2; also see [34]).

Traditionally, studies of microbial succession during leaf litter decomposition have examined the early stages of decomposition for one species. For example, Poll et al. [10] found that bacteria dominated carbon mineralization during the first two weeks, and then fungal decomposers became more abundant on rye (*Lolium perenne*) litter. McMahon et al. [14] used ^13^C-labelled PLFAs to show similar declines in bacterial dominance of litter decomposition over 80 days of ryegrass (*Lolium perenne*) decomposition. However, Wilkinson et al. [13] found that spruce litter became more dominated by bacteria as decomposition progressed. In contrast to our second hypothesis, we found that fungal decomposers dominated decomposition throughout the experiment, though bacterial colonization of litter increased 2–3 fold from the 10 month to the 27-month stage of decomposition. For both single and mixed species litter types, fungal-to-bacterial ratios declined after 27 months in the field, driven by an increase in bacteria abundance, which is likely due to decreasing litter C:N ratios through time [55]. The different microbial colonization progression we see, compared to the studies described above, may be driven by the much-longer (>2 years) duration of the present study, which may reveal a different stage of microbial community development than that investigated in other studies. Because we first sampled after 10 months in the field, we may have missed initial bacterial dominance of litter when soluble compounds were abundant. In a similar study in deciduous hardwood forest, Ball et al. [2] found a decrease in fungal-to-bacterial ratios on some leaf litter mixtures over two years, though both fungal and bacterial biomass initially increased (peaked around 200 days) and then declined. Another possible explanation for the initial fungal dominance of decomposition in our system is that fungal endophytes had ready access to litter and became saprophytic [9], [56], [57]. However, we did not measure fungal endophytes at the outset of this experiment and thus cannot assess their contribution to decomposition in this system. Finally, we performed this study in a high elevation forest (about 2700 m) in Arizona, so it is likely that both temperature and moisture limited decomposition rates and perhaps bacterial colonization in this system, as indicated by mass losses of only 25–45% after more than two years *in situ*. Due to either this slow decomposition rate or our widely spaced sampling intervals, it is possible that we are not capturing the same microbial dynamics found in the above-mentioned other studies. However, if we assume initial PLFA to be negligible, the rate of increase of PLFA concentrations seemed to be declining after 27 months, suggesting that we captured some of the most dynamic interval of microbial community development.

In accordance with our third hypothesis, microbial community composition of leaf litter changed due to both stage of decomposition and litter mixing (Figure 2). Bacterial biomarkers such as *a*15∶0 and *cy*17∶0 showed different abundances on single vs. mixed litterbags through time, perhaps indicating altered colonization by gram-positive (*a*15∶0) and gram-negative (*cy*17∶0) bacteria due to altered substrate availability or competitive dynamics when litters are mixed. The biomarker *a*15∶0 was highly correlated with principle component 1 in our PCA analyses (r^2^ = 0.54, p<0.0001 at 10 months and r^2^ = 0.76, p<0.0001 at 27 months of decomposition), perhaps rendering gram-positive bacteria an interesting target for further examination of microbial decomposer dynamics through time. Bray et al. [43] recently found that microbial community composition determined decomposition rate of single litters at later stages of decomposition rather than earlier stages. Since our study focused on later stages of decomposition (10 months and 27 months), the large changes in microbial community composition we see may also be important for regulating decomposition dynamics when litter has become more recalcitrant. Though we see fungal and bacterial biomass becoming more equivalent between single and mixed litter after 27 months, the makeup of the decomposer community still differs at this stage (Fig. 2). These changes in the decomposer community composition may contribute to the lower, but still significant synergisms seen at this stage of litter decomposition (Table 2).

We hypothesized that total PLFA and fungal-to-bacterial biomass ratios would correlate positively with increasing mass loss of both single and mixed species litter, indicating increasing decomposition with more microbial biomass and a shift towards fungal decomposers. Our fourth hypothesis was only partially supported as higher rates of single litter decomposition rate were positively correlated with total PLFA at 10 months (Fig. 3); yet similar significant correlations were not found for F:B ratios or for any PLFA parameters at 27 months. Single litter decomposition is often regulated by abiotic factors such as microclimate and chemical quality [58], [59]. Thus far, the commonly found stimulations in decomposition resulting from litter mixing have not been readily explained by these factors [20], [21], [23]. A large stimulation in PLFA coincided with a large stimulation in litter decomposition in mixed vs. single species litters (Table 2), suggesting, at least qualitatively, a microbial contribution to non-additive litter decomposition. Yet, we did not find direct significant correlations between total PLFA/F:B and litter mass loss (Fig. 3). Perhaps, in addition to abiotic factors and potential nutrient transfer between litter types, functionally distinct communities of decomposers drive differences in decomposition rate between mixed and single litters. By exploring microbial communities using targeted ribosomal genes [37], [38], we could determine microbial community structure with more resolution. This resolution could allow a better understanding of the abundance of certain functional groups of decomposers and perhaps more explicitly link decomposer identity to decomposition. Manipulative experiments could also provide a better understanding of the relationship between microbial structure and leaf litter decomposition. For example, using fungicides to knock out fungal decomposers could help assess their contribution to decomposition and nutrient release (*sensu* [*60*]).

### Conclusions

Our findings suggest that decomposer communities can change due to shifting plant diversity, although eventual convergence in bacterial and fungal biomass may occur after litter chemistry has been homogenized. Increases in microbial abundance and shifting F:B ratios did not, however, significantly correlate with litter mass loss. Therefore, the prevalence of higher synergisms that occurred earlier during litter decomposition in this system may be due to a combination of microbial community structure changes with other factors. In conclusion, more cogent functional connections between microbial communities and litter decomposition are needed to better understand soil communities and the carbon and nutrient cycling ecosystem services they provide.
